# Discovery of small-molecule inhibitors for the protein-protein interactions involving ATG5

**DOI:** 10.1080/27694127.2023.2215617

**Published:** 2023-05-27

**Authors:** Honggang Xiang, Renxiao Wang

**Affiliations:** Department of Medicinal Chemistry, School of Pharmacy, Fudan University, 826 Zhangheng Road, Shanghai 201203, People’s Republic of China

**Keywords:** Autophagy inhibitor, autophagy-related 5, autophagy-related 16-like 1, binding assay, flow cytometry, western blot, structure-activity relationship

## Abstract

The autophagy-related 12 (ATG12)–autophagy-related 5 (ATG5)–autophagy-related 16-like 1 (ATG16L1) ternary complex forms a dimer that facilitates the translocation of autophagy-related 8 (ATG8) proteins from autophagy-related 3 (ATG3) to phosphatidylethanolamine (PE). This event is fundamental for cargo sequestration and autophagy progression. Thus, one possible strategy for inhibiting autophagy is to disrupt the critical ATG5-ATG16L1 interaction during this process. So far very few known specific autophagy modulators can block autophagy effectively. We recently discovered a small-molecule compound, T1742, which is able to block the ATG5-ATG16L1 and ATG5-TECAIR interactions *in vitro* at the low-micromolar range (IC_50_ = 1~2 μM). Flow cytometry assay and western blot experiments indicated that T1742 can also effectively inhibit autophagy in living cells in a dose-dependent manner. To the best of our knowledge, T1742 represents the first small-molecule autophagy inhibitor that disrupts the protein-protein interactions involving ATG5. Such compounds may serve as a new chemical tool for deciphering the mechanism of autophagy or a potential candidate for therapeutic application.

Over the past few decades, it has been established that autophagy has context-dependent roles in cancer. On the one hand, autophagy prevents cancer progression by killing cancer cells. On the other hand, once cancer is established, increased autophagic flux often enables cancer cells to survive and grow. In the latter situation, abundant preclinical evidences have proven that autophagy inhibition can be applied to cancer treatment. However, most known autophagy inhibitors do not function by a specific mechanism. For instance, chloroquine (CQ), hydroxychloroquine (HCQ), and Bafilomycin A1 (BafA1) inhibit autophagy by blocking lysosomal functions, while they also affect other cellular pathways, including endocytosis and certain vesicular transport routes. Thus, there is still a major gap between the therapeutic potential of modulating autophagy and achievable clinical benefits. To overcome this gap, it is necessary to develop specific autophagy modulators that can not only shed light on the mechanisms of autophagy, but also be used as autophagy-oriented therapies.

The formation of the autophagosomes is a central part in the mechanism of autophagy. During this process, ubiquitin-like autophagy-related 12 (ATG12) is covalently linked to autophagy-related 5 (ATG5) through reactions catalyzed in succession by autophagy-related 7 (ATG7) and autophagy-related 10 (ATG10). The resulting ATG12–ATG5 conjugate then interacts with autophagy-related 16-like 1 (ATG16L1) to form the ternary ATG12–ATG5-ATG16L1 complex. The dimer of this complex, located on the outer membrane of autophagosomes in mammals, acts as an E3-like enzyme to promote lipidation of members of the autophagy-related 8 (ATG8) ubiquitin-like protein family. Lipidated ATG8 plays a critical role in both autophagosome formation, closure and fusion with lysosomes, as well as selective cargo recognition during autophagy (Figure 1). Moreover, ATG12–ATG5 also interacts with tectonin β-propeller repeat containing 1 (TECPR1) in autophagosome–lysosome fusion. Since the ATG12–ATG5-ATG16L1 complex is a key component of the autophagic machinery, we hypothesized that small-molecule compounds that impair its function may inhibit autophagy. The crystal structures of the ATG5-ATG16L1 complex (PDB entry 5NPW) and the ATG5-TECAIR complex (PDB entry 4TQ1) reveal that ATG5 hosts an α-helical segment on ATG16L1 or TECAIR in a long cavity on its surface, where “hot-spot” residues exist in clusters. This α-helical-mediated protein-protein interaction interface is similar to that observed in BCL-2 and MDM2, which have been recognized as “druggable” targets for developing small-molecule anticancer drugs. Mutational studies have demonstrated that disruption of the ATG5-ATG16L1 interaction negatively impacts ATG8-PE formation. Therefore, interfering with the ATG5-ATG16L1 interaction represented a putative strategy to inhibit autophagy.

**Figure 1.**
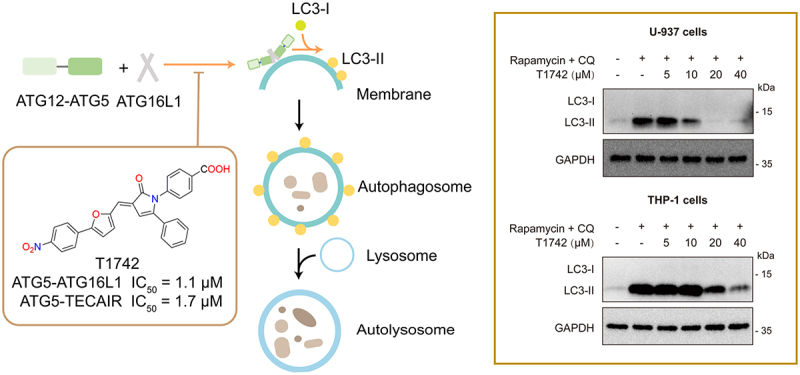
The ATG12–ATG5 conjugate interacts with ATG16L1 to form a ternary complex, which is responsible for ATG8/LC3 lipidation. T1742 is a small-molecule compound that blocks the ATG5-ATG16L1/TECAIR interactions at a low micromolar range (IC_50_ = 1~2 μM) and exhibits a dose-dependent impact on the LC3B-II level in living cells.

In pursuit of this objective, recently we conducted a random screening of our in-house compound collection. Two compounds, LifeChemicals F1418-0361 and TargetMol T8689, were identified since displaying a modest binding affinity toward ATG5 in the homogeneous time-resolved fluorescence (HTRF) binding assay. We subsequently conducted a 2D-structural similarity search using these two hits as queries, which led to the identification of T1742 as a potential blocker of the protein-protein interactions involving ATG5 at the micromolar level, i.e., IC_50_ = 1.1 μM on ATG5-ATG16L1 and IC_50_ = 1.7 μM on ATG5–TECAIR (Figure 1). To validate the inhibitory effect of T1742 on autophagy in living cells, we monitored the LC3B-II expression level by flow cytometry. Indeed, we observed a dose-dependent shift in the LC3B-II level after T1742 treatment. Western blot analysis confirmed that T1742 treatment caused a significant decrease in the LC3B-II level in COS-7, U-937 and THP-1 cells. These findings collectively suggest that T1742 can downregulate autophagy by inhibiting the conjugation of ATG8 proteins to PE.

We chose T1742 as the lead compound to investigate the structure-activity relationship by optimizing its structure, and three sets of derivatives were successfully synthesized. Although we did not obtain any compound with greater potency than T1742, several compounds, such as 12a and 24l, demonstrated comparable efficacy in both *in vitro* binding and cellular assays. It is also encouraging to observe the consistency between the active compounds’ binding assay results and flow cytometry results. Moreover, to understand the structure-activity relationship of T1742 and its derivatives, its possible binding mode to ATG5 was derived through sophisticated molecular dynamics simulations. The outcomes of our simulation helped to interpret the critical role of the carboxyl group on this molecule.

The highlight of our work is the discovery of the first small-molecule ATG5 inhibitor, which exhibits low micromolar affinity to ATG5 in an *in vitro* binding assay and inhibits autophagy in living cells. Although the precise mechanism of action of this compound, as well as its derivatives, is yet to be fully elucidated, our work strongly suggests that small molecules blocking the protein-protein interactions involving ATG5 could be a new class of effective autophagy inhibitors. Such compounds may prove useful as chemical tools to decode the mechanism of autophagy or even have therapeutic implications. We expect the development of new small-molecule ATG5 inhibitors in the future^1^.

## Abbreviations

Autophagy-related 3 (ATG3)

Autophagy-related 5 (ATG5)

Autophagy-related 7 (ATG7)

Autophagy-related 8 (ATG8)

Autophagy-related 10 (ATG10)

Autophagy-related 12 (ATG12)

Autophagy-related 16-like 1 (ATG16L1)

Bafilomycin A1 (BafA1)

Chloroquine (CQ)

Homogeneous time-resolved fluorescence (HTRF)

Hydroxychloroquine (HCQ)

Phosphatidylethanolamine (PE)

Tectonin β-propeller repeat containing 1 (TECPR1)
